# Assessment of Heavy Metal Pollution in Topsoil around Beijing Metropolis

**DOI:** 10.1371/journal.pone.0155350

**Published:** 2016-05-09

**Authors:** Ranhao Sun, Liding Chen

**Affiliations:** Research Center for Eco-Environmental Sciences, Chinese Academy of Sciences, Beijing, People's Republic of China; Sun Yat-Sen University, CHINA

## Abstract

The topsoil around Beijing metropolis, China, is experiencing impacts of rapid urbanization, intensive farming, and extensive industrial emissions. We analyzed the concentrations of Cu, Ni, Pb, Zn, Cd, and Cr from 87 topsoil samples in the pre-rainy season and 115 samples in the post-rainy season. These samples were attributed to nine land use types: forest, grass, shrub, orchard, wheat, cotton, spring maize, summer maize, and mixed farmland. The pollution index (PI) of heavy metals was calculated from the measured and background concentrations. The ecological risk index (RI) was assessed based on the PI values and toxic-response parameters. The results showed that the mean PI values of Pb, Cr, and Cd were > 1 while those of Cu, Ni, and Zn were < 1. All the samples had low ecological risk for Cu, Ni, Pb, Zn, and Cr while only 15.35% of samples had low ecological risk for Cd. Atmospheric transport rather than land use factors best explained the seasonal variations in heavy metal concentrations and the impact of atmospheric transport on heavy metal concentrations varied according to the heavy metal types. The concentrations of Cu, Cd, and Cr decreased from the pre- to post-rainy season, while those of Ni, Pb, and Zn increased during this period. Future research should be focused on the underlying atmospheric processes that lead to these spatial and seasonal variations in heavy metals. The policymaking on environmental management should pay close attention to potential ecological risks of Cd as well as identifying the transport pathways of different heavy metals.

## Introduction

Excessive accumulation of heavy metals is not only toxic to animals and plants [1, 2], but can also lead to elevated heavy metal intake in humans through the food chain [3]. The topsoil serves as the transfer interface for the accumulation of heavy metals in the air, plant, and water. The seasonal and spatial variations of heavy metals in the topsoil are attracting major concern across the world [4].

The concentration of heavy metals in the topsoil is naturally determined by parent materials and subsequent pedogenic processes. However, the anthropogenic input of heavy metals is becoming the main source of heavy metals in the soils of many regions [5–7]. Agrochemicals and organic manure can bring additional heavy metals into the topsoil in agricultural land [8–9]. The heavy metal accumulation in agricultural land is associated with land use types and crop rotation types [10]. Heavy metal sources in urban regions are different from those in agricultural areas. Heavy metals in urban regions are mainly derived from industrial emissions, vehicle exhaust, and the weathering of building and pavement surfaces [11, 12]. The concentration of heavy metals in the topsoil is also impacted by rain washing, leaching, dissolution, volatilization, vegetation uptake, and other associated processes [13, 14]. The accumulation of heavy metals in the topsoil varies according to heavy metal properties and climatic conditions [7, 15]. Some studies have demonstrated that the solubility and mobility of heavy metals are significantly correlated with the physical and chemical properties of soils [16, 17]. Moreover, atmospheric transport plays an important role in the distribution of heavy metals in many regions [18]. For example, the heavy metals transferred by the atmosphere are one to three orders of magnitude higher than those transported through local natural processes [3]. The above studies have shown that multiple causes can affect the accumulation of heavy metals over a large region. Identifying the distribution and variation of heavy metals is essential to reveal underlying processes and to support specific policies in environmental management.

In developing countries such as China, there are many problems associated with heavy metal pollution due to the rapid urbanization and intensive anthropogenic activities [19, 20]. The urban agglomeration around Beijing metropolis is extremely important to China because of its total land area, population, and economy. Geographically, this region is mainly located within the Haihe River Basin (HRB), the largest watershed in the northern China. The HRB covers an area of approximately 318,000 km^2^, including Beijing, Tianjin, and parts of the provinces of Hebei, Shandong, and Shanxi. The total population of the region in 2010 was approximately 146 million, accounting for 10.7% of the nation’s entire population. As one of China’s most important food bases, the HRB produced 10% of the total grain yield, despite only occupying 3.3% of the nation’s overall land area [21]. The HRB combines highly urbanized regions and intensively cultivated farmlands in China. Therefore, there are various sources of heavy metals in the HRB region because of the region’s diverse anthropogenic activities.

The long-term impairment from heavy metal pollution in this region is regarded as a serious danger to mankind, resulting in the “cancer villages” around Beijing reported by China Central Television (CCTV) in 2013 [22, 23]. The adverse effects of heavy metals are usually investigated in large cities such as Beijing and Tianjian [24–28] as well as in some important wetlands and rivers [8, 29]. Few studies have been conducted to reveal the pollution level of heavy metals in the context of the entire basin. Furthermore, studies are mainly focused on the heavy metal risks from crops to human health [30] and potential pollution of heavy metals from farmland soils in the eastern plain of the HRB [31]. The variation in heavy metal concentrations associated with different land use types has received little attention and further assessment is needed.

The objective of this study is to: investigate the pollution levels and ecological risks associated with Cu, Ni, Pb, Zn, Cd, and Cr around Beijing metropolis; and assess the impact of land use types and crop rotations on the variations of heavy metals during the pre- and post- rainy seasons.

## Materials and methods

### Study area

As one of the largest watersheds in China, the HRB is located at 35°3′23″N—42°43′48″N and 111°58′4″E—119°49′15″E. The HRB region has a warm-temperature climate [32] with a mean air temperature of 18°C in May and 22°C in August (our sampling season) [33]. This region is characterized as a typical continental monsoon climate resulting from the dominant impacts of East Asian monsoons. The mean annual precipitation is 509.9 mm and is predominantly concentrated in the short rainy season from June to August. The mean precipitation of the HRB is 30, 75, 150, and 130 mm in May, June, July, and August, respectively [33]. Plateaus, mountains, and alluvial plains contribute to the varied topography around the Beijing metropolis. The mountains of Yanshan and Taihangshan and the loess plateaus are located in the northern and western parts of the HRB, whereas the hilly regions and the alluvial and coastal plains are located in the central and eastern parts. On the plateaus, the dominant vegetation types are natural meadow and cultivated crops. In the mountains, the dominant vegetation types are natural shrubs and coniferous and deciduous forests. The plains support cultivated crops and the urbanized landscape.

We selected sampling sites selected based on the representativeness of the land use types and topographic features as well as the ease of access. No specific permissions were required for these locations and activities in the field sampling and we confirmed that the field studies did not involve any threat to endangered or protected species.

### Soil sample collection

**Fig 1:**
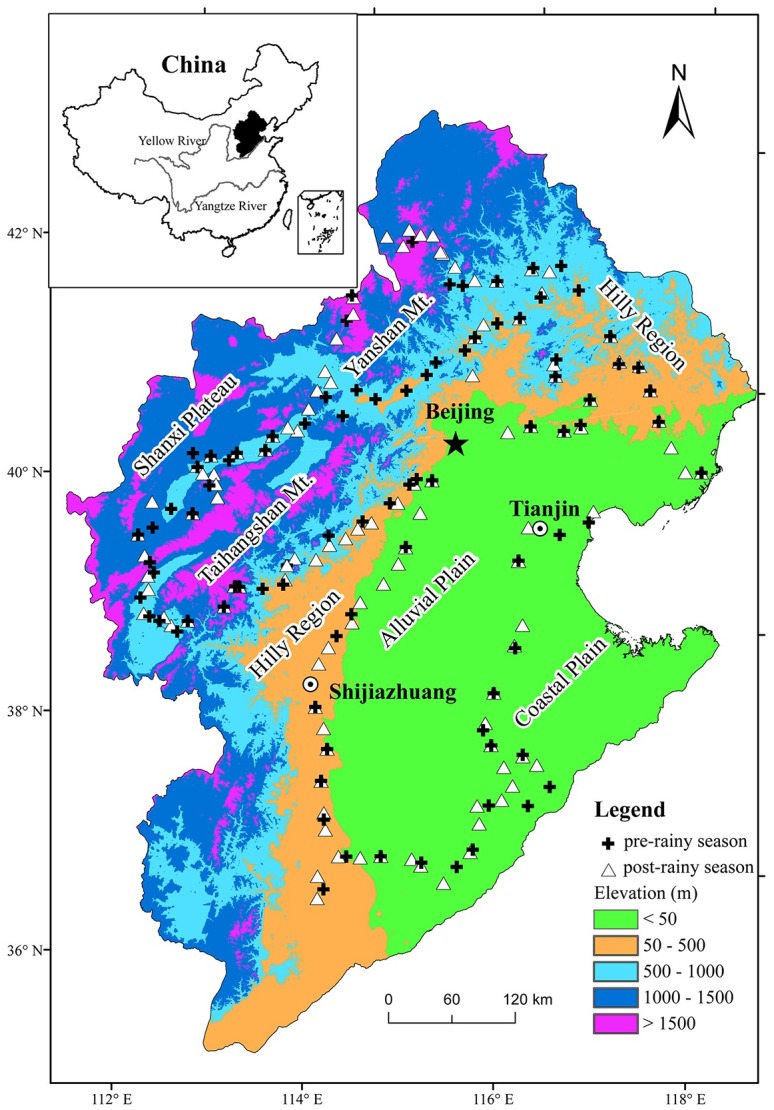
Location of the research region and sampling sites. (The China map was generated using free, open access data sources from the National Geomatics Center of China)

A total of 202 topsoil samples (0~20 cm) were collected in May and August of 2010, representing the pre- and post-rainy season. The specific sampling sites were selected away from the main roads in an attempt to reduce the vehicle impact. Five samples were taken around the center of each site after removing the plant residues using a spade. The samples were mixed thoroughly to select 1000 g of soil as the representative sample for the site and stored in a plastic bag. We recorded the site features including coordinates, altitude, land use type, and topography. In the pre-rainy season, 87 samples were collected from different land use types including forest (21 samples), shrub (9), grass (8), spring maize (12), orchard (12), wheat (20), and mixed farmland (5). In the post-rainy season, 115 samples were selected from the forest (25), shrub (15), grass (9), summer maize (42), orchard (10), cotton (7), and mixed farmland (7). The mixed farmland was used for planting quick-growing crops in mixed patterns, such as beans, potatoes, and peanuts. Wheat was the staple crop and was rotated in one year by the summer maize or the cotton. The spring maize was a single-season crop in the northern China. We examined eight combinations of land use comparison and crop rotations from the pre- to post-rainy season as follows: forest—forest, grass—grass, shrub—shrub, orchard—orchard, mixed farmland—mixed farmland, spring maize—summer maize, wheat—summer maize, and wheat—cotton. The samples covered most of topographic features and land uses around the Beijing metropolis (Fig 1). Road data (grades 1–5), derived from the 1: 250,000 national data set, were used to assess the vehicle impact on heavy metal concentrations. The Euclidean distance was calculated between each sampling site and its nearest road.

### Soil chemical analyses

The soil samples were first air-dried, crushed, and sieved through a 1 mm mesh at room temperature. The soil pH was determined using a standard pH electrode based on 10 g dried soil in 25 ml distilled water. The sieved samples were passed through a 0.15 mm nylon sieve for chemical analysis using Method 3050B (USEPA 1996) [11, 27, 34]. A small portion of each sample (0.25 g) was weighed into a 50 ml Teflon beaker in which a mixture of concentrated HF-HCLO_4_-HNO_3_ (5:5:3) was added. The samples were heated on a hot plate at 200°C and kept at a slight boiling state until the solid residue disappeared [35]. The Cd concentration was determined through inductively coupled plasma mass spectrometry (ICP-MS, Baird ICP2070, USA). The concentrations of Cu, Ni, Pb, Zn, and Cr were determined using an inductively coupled plasma optical emission spectrometer (ICP-OES, Baird ICP2070, USA). The quality assurance and control procedures were implemented by the analysis of a standard reference material obtained from Center for National Standard Reference Material of China. Reagent blanks were monitored throughout the analysis and were used to correct the analytical results. Recovery rates obtained for the studied elements were as follows: Cu = 84.2%, Ni = 88.6%, Pb = 96.5%, Zn = 100.6%, Cd = 92.1%, and Cr = 106.5%.

### Assessment method of pollution and ecological risk

The pollution index (PI) associated with heavy metals can be assessed using the measured and background concentrations [1, 28, 36]. The regional background concentrations of heavy metals were measured by the China National Environmental Monitoring Center in 1980s [25–27, 37]. The PI was calculated by the following equation:

$$PI^{i}=C_{measure}^{i}/C_{background}^{i}$$ (1)

where *$C_{measure}^{i}$* is the measured concentration (mg/kg) of heavy metal *i*, and $C_{background}^{i}$ is the background concentration (mg/kg) of heavy metal *i* in each province of China. A PI value of heavy metals > 1 indicates the accumulation and potential pollution of heavy metals in the topsoil.

Ecological risk index (RI) was used to assess the potential harm caused by heavy metals in the topsoil [36]. The RI was calculated by the following equation:

$$RI^{i}=T^{i}\times PI^{i}$$ (2)

where *T*^*i*^ is the toxic-response parameter (Cd = 30, Cu = Pb = Ni = 5, Cr = 2, Zn = 1) [35, 36]. The ecological risk is ranked into four classes, including low ecological risk (RI < 40), moderate ecological risk (40 ≤ RI < 80), severe ecological risk (80 ≤ RI < 160), and serious ecological risk (RI ≥ 160).

Pearson correlation coefficients were calculated to quantify the relationship among the physical properties and heavy metal concentrations. Analysis of variance was used to test difference in PI between different land use types and different seasons. This analysis indicates that the PI difference is reliable when the significance test is true (*p* < 0.05). All statistical analyses were conducted using SPSS 19.0 (SPSS Inc., USA).

## Results and Discussion

### Physical and chemical properties of samples

This study was implemented in a large basin topography characterized by various natural and anthropogenic features. Elevation of sampling sites ranged from 6 to 1800 m with an average of 509 m above sea level. The soil pH ranged from 6.6 to 8.3 with an average of 7.6. The mean distance of sampling sites to the nearest road was 1.04 km with a range from 0.005 to 24.79 km. The natural land uses (forest, grass, shrub) were located at high elevations while cultivated land uses (wheat, cotton, orchard, mixed farmland, and spring and summer maize) were located at low elevations (Table 1).

**Table 1:** Physical and chemical properties (mean ± SD) of soils in nine land use types.

land use		Physical properties			Chemical properties (mg/kg)					
	N	Elevation (m)	Soil pH	Distance from nearest road (m)	Cu	Ni	Pb	Zn	Cd	Cr
Forest	46	667±71	7.45±0.07	1464±655	23.03±1.41	18.15±1.06	23.84±1.2	63.17±4.52	0.218±0.015	79.02±4.67
Grass	17	1029±118	7.61±0.12	1807±1244	22.29±2.07	18.56±1.58	18.02±1.17	45.71±5.17	0.152±0.013	74.38±7.15
Shrub	24	653±74	7.64±0.1	1416±836	23.42±1.81	20.54±1.7	23.45±1.41	61.73±5.52	0.193±0.014	77.17±4.71
Orchard	22	584±100	7.51±0.11	1187±392	28.8±2.49	19.04±1.45	23.91±1.56	71.06±9.92	0.223±0.021	79.4±5.45
Wheat	20	26±6	7.55±0.1	507±124	26.46±1.08	18.69±0.83	21.31±0.68	57.2±2.87	0.247±0.017	103.83±2.62
Cotton	7	38±8	7.8±0.15	86±17	26.11±3.07	26.66±2.12	28.6±1.78	72.53±7.82	0.183±0.008	76.19±6.74
Mixed farmland	12	639±114	7.57±0.11	580±1480	30.41±4.07	21.01±1.57	22.34±1.31	54.68±5.24	0.196±0.02	84.15±6.82
Spring maize	12	784±85	7.49±0.1	387±205	29.08±2.31	16.64±1.13	17.53±1	70.57±15.45	0.262±0.04	97.86±3.23
Summer maize	42	223±55	7.73±0.08	255±74	28.83±1.54	26.61±0.82	29.28±1.32	71.53±3.71	0.202±0.015	71.41±2.63

N refers to the sampling number.

Results of Pearson correlation analysis showed that the elevation was significantly correlated with the chemical properties of sampling sites (Table 2). The soil pH had negative relationships with Cd and Cr concentrations while the nearest road distance had negative relationships with Cu and Cr concentrations. The correlation matrix showed that strong relationships were found among the concentrations of Ni, Pb, and Zn. The Cu concentration was found to have a strong positive correlation with that of Cr. The Cd concentration had lower relationships with other heavy metals.

**Table 2:** Correlation coefficients among physical and chemical properties of samples.

	Elevation	Soil pH	Distance from nearest road	Cu	Ni	Pb	Zn	Cd
Soil pH	0.13							
Distance from nearest road	0.33**^+^	0.01						
Cu	-0.28**	-0.09	-0.24**					
Ni	-0.21**	0.17	-0.09	0.24**				
Pb	-0.39**^+^	-0.04	-0.03	0.22**	0.33**^+^			
Zn	-0.25**	-0.03	-0.10	0.29**	0.37**^+^	0.43**^+^		
Cd	-0.24**	-0.21**	-0.09	0.26**	-0.06	0.26**	0.23**	
Cr	-0.25**	-0.20**	-0.25**	0.45**^+^	0.10	-0.13	0.27**	0.25**

** p < 0.01;

**^+^ the strong relationship with correlation coefficient > 0.3.

### Spatial variations in heavy metal concentration

**Fig 2:**
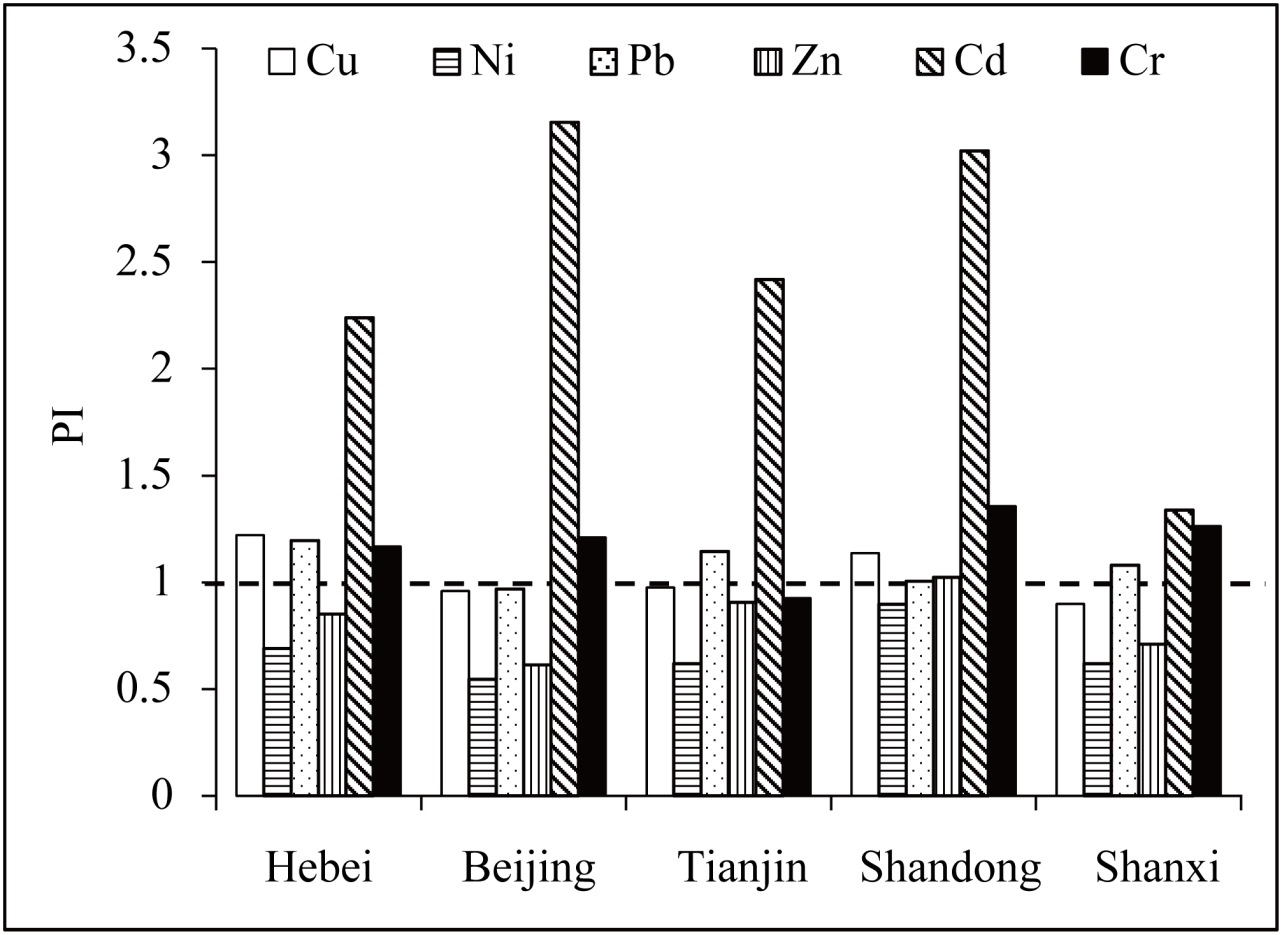
Pollution indices (PI) of heavy metals in different administrative regions.

The measured concentrations of heavy metals were compared with the background concentrations and the China environmental quality standard for soils (EQSS) (Table 3). The EQSS was ranked into two grades [37]. The first grade of EQSS is to meet the needs of natural protection while the second grade is to satisfy the needs of agricultural production and human health [38]. Although only Cd concentrations were below the first grade of EQSS, some of the measured concentrations exceeded the background values of heavy metals (Fig 2). We identified four heavy metals posing pollution risks in Hebei Province, including Cu, Pb, Cd, and Cr. Three heavy metals posed pollution risks in the provinces of Shandong (Cu, Cd, and Cr) and Shanxi (Pb, Cd, and Cr). Only two heavy metals were found to be associated with pollution risks in Beijing (Cd and Cr) and Tianjin (Pb and Cd).

**Table 3:** Heavy metal concentrations (mg/kg) of measurement (MV), background (BV), and environmental quality standard for soils (EQSS).

		Cu	Ni	Pb	Zn	Cd	Cr
First grade of EQSS	Mean	35	40	35	100	0.2	90
Second grade of EQSS	Mean	100	60	350	300	0.6	250
BV of Hebei	Mean	21.8	30.8	21.5	78.4	0.094	68.3
BV of Beijing	Mean	23.6	29	25.4	102.6	0.074	68.1
BV of Tianjin	Mean	28.8	33.3	21	79.3	0.09	84.2
BV of Shandong	Mean	24	25.8	25.8	63.5	0.084	66
BV of Shanxi	Mean	26.9	32	15.8	75.5	0.128	61.8
BV of China	Mean	22.6	26.9	26	74.2	0.097	61
MV of Hebei	Mean	26.63	21.29	25.74	66.83	0.211	79.72
N = 121	Range	6.33–67.86	1.84–39.01	10.26–49.89	14.06–246.05	0.049–0.644	21.70–154.80
	SD	10.32	7.24	7.88	30.98	0.09	28.19
MV of Beijing	Mean	22.64	15.82	24.62	63.04	0.233	82.46
N = 13	Range	11.19–33.31	5.77–26.85	15.47–45.37	20.68–142.00	0.132–0.590	43.20–107.70
	SD	7.2	6.54	7.95	31.96	0.12	19.41
MV of Tianjin	Mean	28.12	20.62	24.04	71.96	0.218	78.06
N = 8	Range	13.46–45.76	7.09–31.04	11.35–30.65	12.22–137.59	0.125–0.416	32.00–133.20
	SD	12.06	8.01	6.88	43.8	0.09	32.53
MV of Shandong	Mean	27.28	23.19	25.94	65.05	0.254	89.49
N = 21	Range	21.08–38.54	15.20–34.62	16.00–37.27	35.44–101.47	0.145–0.753	52.41–113.20
	SD	5.5	6.08	6.28	19.41	0.14	14.43
MV of Shanxi	Mean	24.19	19.84	17.07	53.73	0.171	78.01
N = 39	Range	8.98–76.74	6.51–39.07	10.80–25.69	22.05–227.60	0.108–0.370	49.16–118.00
	SD	11.09	6.96	3.35	31.02	0.05	21.05
MV of HRB	Mean	26.03	20.83	23.95	64.07	0.209	80.52
N = 202	Range	6.33–76.64	1.84–39.07	10.26–49.89	12.22–246.05	0.049–0.753	21.70–154.80
	SD	10	7.17	7.75	30.79	0.09	25.47
	Kurtosis	4.638	-0.282	0.193	8.729	7.902	-0.426
	Skewness	1.51	-0.002	0.692	2.011	2.191	0.137

SD refers to the standard deviation. N refers to the sampling number.

The mean PI of all samples increased in the following order: Ni (0.718) < Zn (0.859) < Cu (0.977) < Pb (1.132) < Cr (1.219) < Cd (2.260). The proportion of sampling sites with a PI > 1 was 9.90%, 25.74%, 35.15%, 61.88%, 64.85%, and 93.07% for Ni, Zn, Cu, Pb, Cr, and Cd, respectively. The RI values of Cu, Ni, Zn, Pb, and Cr were less than 40 for all samples which indicated low ecological risks. However, the RI values of Cd indicated high ecological risks in the region. There were 15.35% of total samples with a low ecological risk, 60.4% of total samples with a moderate ecological risk, 22.28% of total samples with a severe ecological risk, and 1.98% of total samples with a serious ecological risk.

The heavy metal concentrations around the Beijing metropolis have been investigated in many studies (Table 4). Generally, the concentrations of heavy metals were higher in urban regions than in areas with cultivated and natural land use. Previous studies in the Beijing and Tianjin cities have shown that the measured concentration of Cd was higher than the background value [28]. The high concentration of Cd has also been investigated in the plain areas of Hebei Province [31]. A recent investigation indicated that almost 17% of China’s farmlands had suffered from heavy metal pollution; this investigation showed that Cd had the highest ecological risk all over the country [20]. All these results indicated that the Cd pollution has become the most significant heavy metal risk during the industrialization and urbanization of the developing China.

**Table 4:** Mean concentration (mg/kg) of heavy metals in different regions.

Study area	Cu	Ni	Pb	Zn	Cd	Cr	Land use	Reference
Beijing	18.7	26.8	24.6	57.5	0.119	29.8	Natural land	[39]
Beijing	22.4		20.4	69.8	0.136		Suburban agricultural land	[19]
Beijing	22.5	25	24	75.6		61.3	Rural land	[25]
Beijing	34.42	25.87	39.5	89.63	0.192	60.27	Urban land	[27]
Beijing	23.7	27.8	28.6	65.6	0.148	35.6	Urban and rural land	[11]
Beijing and Tianjin	28.2		18.7	71	0.145	52.3	Urban and suburban land	[26]
Beijing and Tianjin	24.5		5.9	97.3	0.46	43.5	Wastewater irrigated cultivated land	[40]
Tianjin	67		52.5	100.6	0.49	101	Suburban land	[24]
Shandong	24.78	29.46	24.37	71.94	0.19	64.41	Cultivated land	[30]
China	30.67	30.7	34.86	85.33	0.25	65.27	Cultivated land	[20]
HRB	26.03	20.83	23.95	64.07	0.209	80.52	Cultivated and natural land	This study
HRB	28.32	22.30	24.97	67.64	0.218	82.72	Cultivated land	This study
HRB	22.99	18.89	22.59	59.36	0.198	77.60	Natural land	This study

### Seasonal variations in heavy metal concentrations

**Fig 3:**
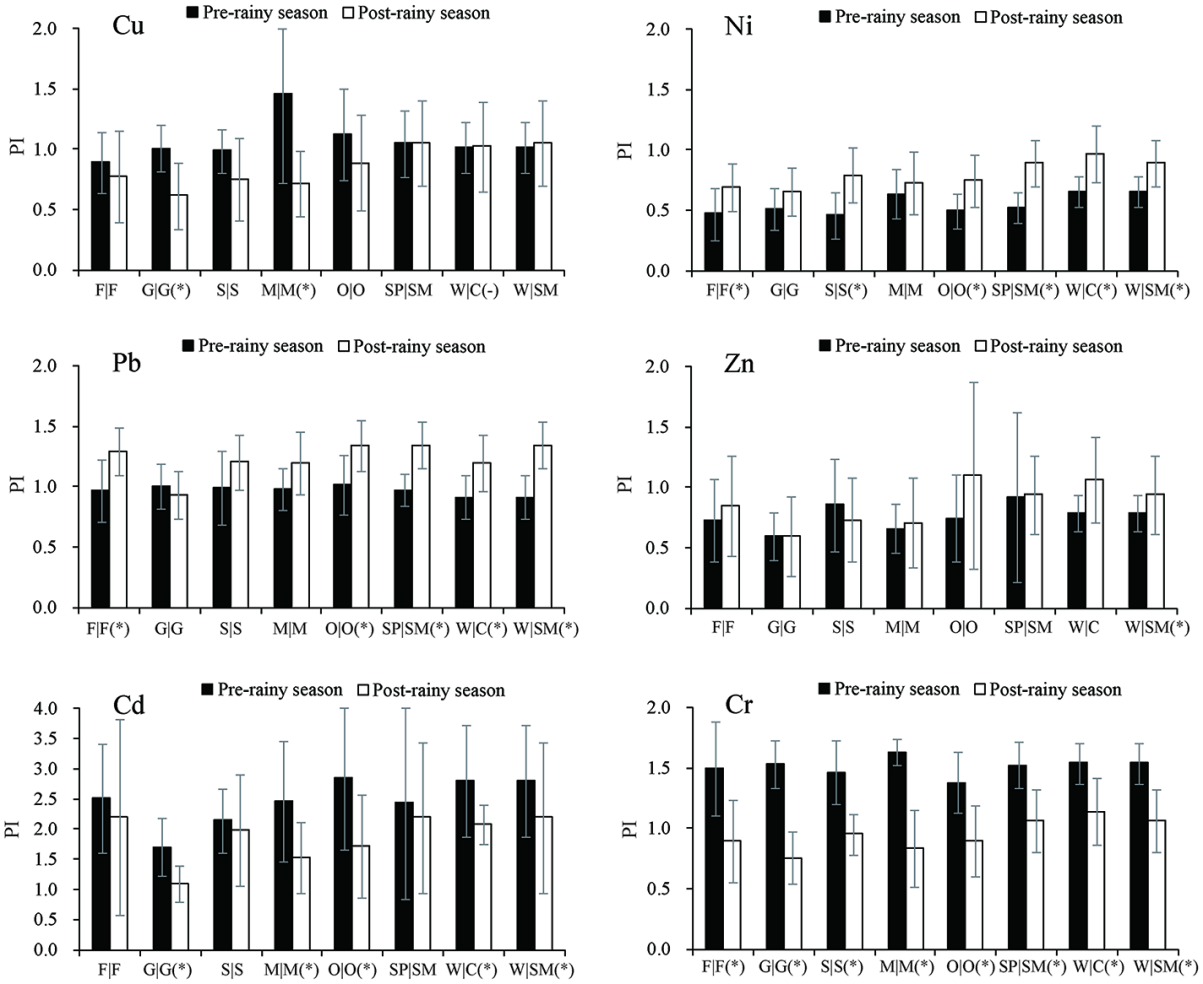
Difference of pollution index (PI ± SD) and statistical test (* *p* < 0.05) between the pre- and post- rainy seasons. F (Forest), G (grass), S (shrub), M (mixed farmland), O (orchard), SP (spring maize), SM (summer maize), W (wheat), and C (cotton).

We compared the mean PI of heavy metals between the pre- and post- rainy seasons. The statistical test (*p* value) of PI difference was calculated to assess the reliability of seasonal variations (Fig 3). We found that about 56% of the total PI comparison pairs (27 pairs) were significantly different (*p* < 0.05). However, the results indicated that seasonal variations varied according to different types of heavy metals. Specifically, most PI values for Cu, Ni, and Zn were < 1, both in the pre- and post-rainy seasons. In contrast, Cd had the highest PI values during this period. The PI values for Cr were > 1 in the pre-rainy season only, while they were > 1 for Pb in the post-rainy season. Overall, the mean PI values of Cu, Cd, and Cr decreased from the pre- to post-rainy season, which was opposite to the trend of the PI values of Ni, Pb, and Zn.

Table 5 showed the statistical significance (*p* value) of the PI difference between each pair of land use types (Table 5). The results showed that only 41 pairs of PI comparisons (about 10% of the total comparisons) were statistically significant (*p* < 0.05). This result indicated that the impact of land use was not significant compared with the impact of precipitation.

**Table 5:** Significance test (*p < 0.05) of pollution index (PI) difference among different land use types.

PI comparison	Significance test of PI difference in pre-rainy season						Significance test of PI difference in post-rainy season					
	Cu	Ni	Pb	Zn	Cd	Cr	Cu	Ni	Pb	Zn	Cd	Cr
Forest *vs* Shrub												
Forest *vs* Grass												
Forest *vs* Orchard				*				*		*		
Forest *vs* Mixed farmland												
Forest *vs* Spring maize												
Forest *vs* Summer maize												
Forest *vs* Wheat						*	*				*	
Forest *vs* Cotton	*											*
Shrub *vs* Grass							*					
Shrub *vs* Orchard								*		*	*	
Shrub *vs* Mixed farmland												
Shrub *vs* Spring maize												
Shrub *vs* Summer maize												
Shrub *vs* Wheat						*			*			
Shrub *vs* Cotton	*											*
Grass *vs* Orchard												
Grass *vs* Mixed farmland				*				*				
Grass *vs* Spring maize										*		
Grass *vs* Summer maize												
Grass *vs* Wheat						*	*	*	*	*	*	
Grass *vs* Cotton	*		*	*								*
Orchard *vs* Mixed farmland						*	*	*	*	*	*	
Orchard *vs* Spring maize												
Orchard *vs* Summer maize												
Orchard *vs* Wheat							*					
Orchard *vs* Cotton	*				*							*
Mixed farmland *vs* Spring maize												
Mixed farmland *vs* Summer maize												
Mixed farmland *vs* Wheat												
Mixed farmland *vs* Cotton												
Spring maize *vs* Wheat												
Summer maize *vs* Cotton	*											*

### Implications for environmental management

The concentrations of Ni, Zn, and Cu were close to their background concentrations. It was therefore expected that there would not be significant pollution risks associated with Ni, Zn, and Cu. However, the pollution index of Pb, Cd, and Cr varied from the pre- to post-rainy seasons. The RI values showed that a large proportion of samples had moderate and high ecological risks associated with Cd. The high level of Cd risks agreed with the results of multiple studies in this region [20, 28, 31]. In addition, previous studies have shown that most elements of Cd were exchangeable and soluble compared with other heavy metals in Hebei Province [31]. The labile fractions of Cd can increase the potential bioavailability and bioaccessibility. The rapid urbanization around the Beijing metropolis expedites the uptake and establishment of metal plating and welding industries in this region. The metal-processing industry is regarded as an important source of anthropogenic Cd and Cr [4]. Management measures should pay more attention to the accumulation of Cd around the Beijing metropolis and industrial activities in relation to Cd should be monitored and controlled in the region.

The impact of land use types and crop rotations on the heavy metal accumulation has been investigated in many regions. Previous studies have shown high concentrations of heavy metals in cultivated land-use types rather than natural land-use types [4, 8–10, 39]. The results of the current study indicated a slight difference in heavy metal concentrations among different land use types (Fig 3). However, the difference in heavy metal concentrations was not significant according to the statistical tests (Table 5). These results indicated that the source of heavy metals in natural lands was similar to that of cultivated lands. One potential explanation for this was that the main source of heavy metals was industrial emissions transferred by atmospheric deposition. Compared with the local input of heavy metals from agricultural activities, the atmospheric deposition extended the distribution of heavy metals from one place to other places on large spatial scales [41]. A recent research has investigated the dustfall from Inner Mongolia, Hebei Province, and Beijing, which served as an important carrier of heavy metals [42]. Therefore, large-scale atmospheric transport may play an important role in the distribution of heavy metals around the Beijing metropolis. Heavy metal risks in natural land areas should be given more attention because of its similarities in pollution levels with cultivated land areas.

The precipitation was an important contributor to atmospheric transport at large spatial scales [18]. The current study described the PI variations of heavy metals from the pre- to post-rainy seasons. Increasing trends in PI values were found in Ni, Pb, and Zn concentrations and declining trends were found in Cu, Cr, and Cd concentrations. In contrast, the precipitation facilitated the leaching and dilution of heavy metals in the topsoil during the rainy season. The precipitation dynamics could impact the accumulation of heavy metals during a specific period. Understanding the climatic processes that have resulted in the redistribution of heavy metals is a key research priority for further studies. Application of a coupled model between regional atmospheric features and industrial emissions may be useful to better understand the relationships in heavy metal concentrations between air, soil, and water.

Although we tried to address the potential limitations of our research, there were some issues that must be fully addressed in future studies. The sampling sites did not cover the topsoil in urban regions that may be more directly impacted by human-induced factors. The topsoil polluted by wastewater irrigation in some farmlands was also not considered in the current study. Recent studies have shown that high levels of heavy metal pollution resulted from wastewater irrigation and the application of wastewater sludge to farmland in some parts of this region [13, 40, 43]. In addition, the measurement of heavy metals was only conducted during a single year in this study. Some studies have shown that seasonal emissions of industry can impact the redistribution of heavy metals within a region [4]. The period of anthropogenic activities may be another potential contributor to the observed seasonal variation in heavy metal concentrations. We suggest that the next challenge is to understand the underlying atmospheric processes that lead to these spatial and seasonal variations of heavy metal concentrations. It is also critical to develop tools and technologies for modeling and predicting future trends of heavy metal accumulation.

## Conclusions

This study investigated the heavy metal concentrations in different land use types around the Beijing metropolis. The pollution levels and ecological risks associated with Cd were found to be higher than other heavy metals in all land use types. We also observed that concentrations of Pb, Ni, and Zn increased from the pre- to post-rainy season while those of Cd, Cr, and Cu decreased during this period. The results of this study indicated that the impact of land use on heavy metal risks was not statistically significant. We inferred that the atmospheric transport may play a key role in the distribution of heavy metals in this region. The further improvement of coupled models of atmospheric processes and industrial emission will be helpful to better understand the accumulation of heavy metals around the Beijing metropolis.

## Supporting Information

S1 Table The concentrations of heavy metals in 202 topsoil samples. https://figshare.com/articles/Supplementary_file_xlsx/3187756
(XLSX)
